# Virtual environments for the transfer of navigation skills in the blind: a comparison of directed instruction vs. video game based learning approaches

**DOI:** 10.3389/fnhum.2014.00223

**Published:** 2014-05-01

**Authors:** Erin C. Connors, Elizabeth R. Chrastil, Jaime Sánchez, Lotfi B. Merabet

**Affiliations:** ^1^The Laboratory for Visual Neuroplasticity, Department of Ophthalmology, Massachusetts Eye and Ear Infirmary, Harvard Medical SchoolBoston, MA, USA; ^2^Department of Psychology, Center for Memory and Brain, Boston UniversityBoston, MA, USA; ^3^Department of Computer Science, Center for Advanced Research in Education, University of ChileSantiago, Chile

**Keywords:** early blind, late blind, navigation, spatial cognition, games for learning, videogames, virtual environment, near transfer of learning

## Abstract

For profoundly blind individuals, navigating in an unfamiliar building can represent a significant challenge. We investigated the use of an audio-based, virtual environment called Audio-based Environment Simulator (AbES) that can be explored for the purposes of learning the layout of an unfamiliar, complex indoor environment. Furthermore, we compared two modes of interaction with AbES. In one group, blind participants implicitly learned the layout of a target environment while playing an exploratory, goal-directed video game. By comparison, a second group was explicitly taught the same layout following a standard route and instructions provided by a sighted facilitator. As a control, a third group interacted with AbES while playing an exploratory, goal-directed video game however, the explored environment did not correspond to the target layout. Following interaction with AbES, a series of route navigation tasks were carried out in the virtual and physical building represented in the training environment to assess the transfer of acquired spatial information. We found that participants from both modes of interaction were able to transfer the spatial knowledge gained as indexed by their successful route navigation performance. This transfer was not apparent in the control participants. Most notably, the game-based learning strategy was also associated with enhanced performance when participants were required to find alternate routes and short cuts within the target building suggesting that a ludic-based training approach may provide for a more flexible mental representation of the environment. Furthermore, outcome comparisons between early and late blind individuals suggested that greater prior visual experience did not have a significant effect on overall navigation performance following training. Finally, performance did not appear to be associated with other factors of interest such as age, gender, and verbal memory recall. We conclude that the highly interactive and immersive exploration of the virtual environment greatly engages a blind user to develop skills akin to positive near transfer of learning. Learning through a game play strategy appears to confer certain behavioral advantages with respect to how spatial information is acquired and ultimately manipulated for navigation.

## Introduction

Considerable interest has arisen regarding the use of virtual reality environments and video games for education, rehabilitation, as well as mental fitness training (Mayo, 2009; Bavelier et al., 2011, 2012; Lange et al., 2012). Moreover, simulation-based training combined with ludic-based approaches for learning have been associated with behavioral gains including the development and reinforcement of sensory, motor, and cognitive skills that might otherwise be more difficult, or even too dangerous, to learn under more typical training settings (e.g., Kuppersmith et al., 1996; Pataki et al., 2012; Rizzo et al., 2012). It has been proposed that realistic and immersive virtual environments allow individuals the opportunity to interact with objects and events in novel and meaningful ways, acquire relevant contextual information, and “integrate knowledge by doing” (Shaffer et al., 2005). Furthermore, the open structure and self-directed discovery of information inherent in these virtual settings improves contextual learning and the transfer of situational knowledge (Shaffer et al., 2005; Dede, 2009). Thus, successfully leveraging these advantages in the education and rehabilitation arenas could have immense appeal by facilitating the learning of demanding tasks and the transfer of acquired skills.

While the exploration of virtual environments and video game play are typically ascribed to the visual modality, another potential application of these approaches could be for the training and rehabilitation of individuals with profound blindness. Blind individuals typically undergo formal instruction referred to as orientation and mobility (O&M) training as a means of learning how to navigate independently through the environment. Unlike the sighted, blind individuals must rely on other sensory channels (such as hearing, touch, and proprioception Thinus-Blanc and Gaunet, 1997) to gather relevant spatial information for orientating, route planning, and path execution (Strelow, 1985; Ashmead et al., 1989; Loomis et al., 1993; Long and Giudice, 2010). The resultant mental representation of the surrounding space is referred to as a spatial cognitive map (Strelow, 1985), and generating an accurate and robust mental map is considered essential for efficient travel (Siegel and White, 1975; Blasch et al., 1997). Not surprisingly, situations where the environment is particularly complex or unfamiliar (or when familiar routes are no longer accessible) can represent a significant challenge when navigating without the benefit of sight. Certainly, many technical advancements and assistive devices have been developed to help blind individuals (including sensory substitution devices, digital maps, and GPS based systems) (e.g., Petrie et al., 1996; Loomis et al., 2005; Johnson and Higgins, 2006; Giudice et al., 2007; Kalia et al., 2010; Chebat et al., 2011; see also Giudice and Legge, 2008 for review). However, many of these approaches are difficult to learn, may require modifications to existing infrastructure, or are not readily adaptable to all situations. Moreover, from a learning and training standpoint, assistive devices are not typically designed for the purposes of training navigation skills.

Based on these observations, we developed an audio-based, virtual environment called Audio-based Environment Simulator (AbES) that can be explored to access contextually relevant spatial information for the purposes of surveying and learning the layout of an unfamiliar complex indoor environment. Key to this user-centered approach is the dynamic and interactive manner in which the spatial information is acquired, which engages the user to construct a spatial cognitive map of a designated space. The contextually relevant spatial information acquired can then be used for the purposes of navigation once the user arrives at the physical environment. This training strategy is comparable to the concept of near transfer of learning, which presupposes that there is a contextual overlap between the training and transfer settings, and that the training content is relevant to the task in question (Cormier and Hagman, 1987).

As a proof of concept (Merabet et al., 2012), we previously demonstrated that early and profoundly blind participants (i.e., documented prior to the age of three) who interacted with AbES were able to create an accurate spatial mental representation that corresponded to the spatial layout of an existing physical building. Furthermore, self-directed exploration carried out within a context of a video game metaphor allowed for the transfer of acquired spatial information for the purpose of navigating through an environment with which they were previously unfamiliar (Merabet et al., 2012).

Here, we present the results of a larger-scale study aimed at comparing the development and transfer of spatial information learned through self-directed game play with a structured, didactic approach. Specifically, we compared the exploration and learning of a virtual environment through either: (1) self-directed exploration and implicit learning under the pretext of a video game metaphor or (2) directed instruction and explicit learning with the aid of a sighted facilitator. Finally, as a control condition, we also compared performance in a subset of participants who learned the spatial layout of a virtual environment following the same video game metaphor. However, in this latter condition, the virtual environment did not correspond to the target physical environment.

Employing this study design allowed for a direct comparison between the mode of interaction (i.e., self-directed, implicit learning through gaming vs. guided instruction, explicit learning through directed navigation) as well as controlling for the effect of contextual information (i.e., playing in a corresponding environment vs. non corresponding environment). By assessing the transfer of spatial information acquired from the exploration of the virtual environment, participants' spatial knowledge regarding the layout of the target building could be ascertained objectively. Given that the participants were completely unfamiliar with the layout of the target building and further, they were never explicitly trained to navigate the routes that were ultimately tested, we would interpret successful navigation performance as evidence of positive near transfer of learning. As a secondary goal, we also investigated the potential association of a number of factors of interest on navigation performance, including prior visual experience, age, gender, and verbal memory ability. The results from a subset of individuals (three) participating in our pilot study (Merabet et al., 2012) were incorporated into the analysis presented here.

Based on the possibility that cognitive behavioral gains may result from video game play, we hypothesized that participants who learned the environment through self-directed exploration would show evidence of transfer of spatial knowledge greater than or equal to those who were explicitly taught the environment through directed navigation. Further, these cognitive gains (i.e., near transfer of learning) would only arise if exploration occurred within an environment that corresponded to the target building where the acquired skills would ultimately be assessed. Secondly, given previous accounts suggesting that prior visual experience may have a beneficial effect on the ability to mentally represent surrounding space (Ashmead et al., 1989), we hypothesized that late blind participants (i.e., individuals having greater prior visual experience) would show a behavioral advantage compared to their early blind counterparts.

## Methods

### Participants and study design

Thirty-eight profoundly blind individuals aged between 18 and 45 years (mean age 27.92 years ± 8.51 *SD*; 20 males, 18 females) participated in the study. Blindness was defined as residual visual function no greater than perceived light perception, hand motion, color, or shadows. The etiology of blindness varied across participants, however all were of ocular related cause (e.g., retinitis pigmentosa, glaucoma, Leber's congenital amaurosis, retinopathy of prematurity). We defined early blind as documented profound blindness acquired prior to the age of three (i.e., typically prior to the development of high level language function and the retention of vivid visual memories). While the majority of the participants had diagnoses that could be considered of congenital cause, we relied on evidence of profound blindness based on documented visual functional assessment. In contrast, late blind was defined as blindness acquired after the age of 14. In this latter group, profound vision loss occurred well after the development of high level language function and all participants had prior visual memories based on self-report. All participants had no other neurological or health concerns, had self-reported normal spatial hearing, and had received formal O&M training prior to participating in the study albeit with varying degrees of experience (mean 8.33 years ± 8.24 *SD*; see Table 1). The majority of the participants were right handed (based on self-report) but all used their right hand to operate the control keys of the software. Five additional participants were excluded from the study prior to any testing due to personal and/or medical reasons (unrelated to their participation in the study) and thus were unable to complete the behavioral assessments. All participants provided written informed consent in accordance with procedures approved by the investigative review board of the Massachusetts Eye and Ear Infirmary (Boston, MA, USA) and all training and performance assessments were carried out at the Carroll Center for the Blind (Newton, MA, USA). As participants had varying levels of residual visual function (e.g., hand motion, light perception), all wore a blindfold throughout the training and behavioral assessment sessions so as to eliminate the possibility of relying on any visual related cues. Participants were allowed to use their cane as a mobility aid during the behavioral assessments if they chose.

**Table 1:** **Participant characteristics separated by study subgroups**.

**Group**	**Sub group**	**Gender**	**Age (years ± *SD*)**	**Verbal memory (score ± *SD*)**	**O&M (years ± *SD*)**
Gamers	Early blind	4 m/4 f	24.00 ± 6.76	81.13 ± 15.29	14.25 ± 7.78
	Late blind	5 m/2 f	29.86 ± 7.34	72.43 ± 18.16	1.0 ± 0.00^*^
	Subgroup totals/means	9 m/6 f	26.73 ± 7.42	77.07 ± 16.69	8.07 ± 8.78
Directed navigators	Early blind	4 m/3 f	28.29 ± 9.05	87.86 ± 6.82	10.43 ± 9.91
	Late blind	4 m/3 f	31.71 ± 10.45	83.43 ± 11.49	1.0 ± 0.00^*^
	Subgroup totals/means	8 m/6 f	30.00 ± 9.56	85.64 ± 9.36	5.71 ± 8.32
Controls	Early blind	2 m/2 f	21.00 ± 2.94	88.75 ± 7.37	14.25 ± 1.71
	Late blind	0 m/3 f	33.33 ± 9.71	89.33 ± 6.03	14.00 ± 5.29
	Subgroup totals/means	2 m/5 f	26.29 ± 8.90	89.00 ± 6.27	14.14 ± 3.29
Totals/means		20 m/18 f	27.92 ± 8.51	82.72 ± 13.25	8.33 ± 8.24

* No more than 1 year of O&M experience.

As potential factors of interest associated with navigation performance, we also collected age, gender, and verbal memory ability (Table 1). Verbal memory recall was assessed using the Wechsler Memory Scale; Third Edition (WAIS-III) Word List Test. For details regarding this assessment see WAIS-III (1997).

**Figure 1:**
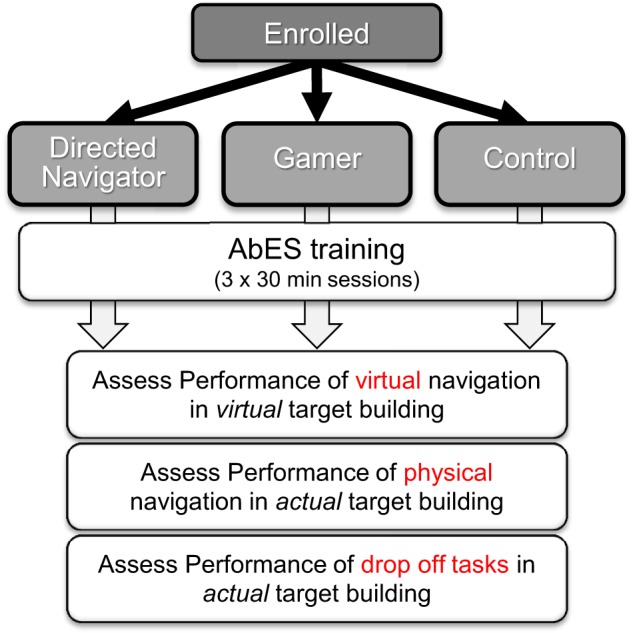
**Overall study design**. Using a stratified randomization strategy, early and late blind participants were relegated to one of three experimental groups; (1) gamers (2) directed navigators, or (3) control. Training included 3, 30-min sessions. Following game play/training, the participants underwent a series of three sequential behavioral task assessments.

Using a stratified randomization strategy (i.e., based on early or late blind status), participants were relegated to one of three experimental groups; (1) gamers (*n* = 15) (2) directed navigators (*n* = 14), or (3) control (*n* = 7) (Figure 1). Training included three, 30-min sessions (for a total of 90 min) plus an initial familiarization period (roughly 10 min) to learn the key strokes and corresponding audio cues used in the software. For participants in the gamer and control groups, the rules and goals associated with game play were also presented. Prior to enrollment, we verified that all study participants were completely unfamiliar with the layout of target building (by formal questioning) as well as to the overall purpose of the study. This was necessary to minimize any potential confounds related to expectation bias and prior experience on the assessments of performance.

**Figure 2:**
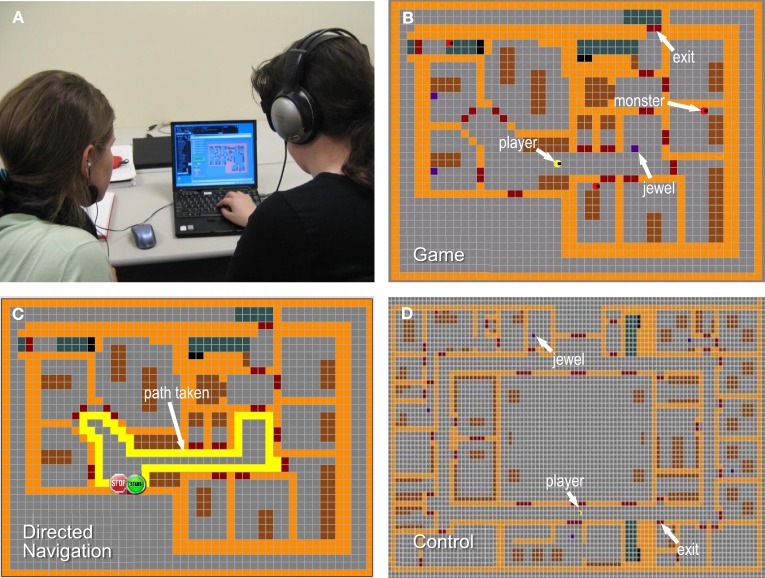
**Virtual rendering of an existing two story building (for simplicity, only the first floor is shown) represented in the AbES software used. (A)** Blind participants (right) interacting with the AbES software while a facilitator (left) looks on. **(B)** In gamer mode, the user (yellow icon) navigates through the virtual environment using auditory cues to locate hidden jewels (blue squares) and avoid being caught by roving monsters (red icons). In directed navigation mode **(C)**, the user learns the spatial layout of the building and the relative location of the rooms using a series of predetermined paths (shown in yellow) with the assistance of a facilitator (for simplicity, only one path is shown here). **(D)** For the control group, the user played in a virtual environment that did not correspond to the target building.

Participants in the gamer group interacted with AbES within the context of a first-person video game designed to promote the full exploration of the virtual environment (Figure 2A). Following a goal-directed strategy, the game's premise is to explore the entire virtual building in order to collect as many jewels as possible (randomly hidden in various rooms) while avoiding roving monsters that are programmed to take away the jewels and hide them in other locations (Figure 2B). Once a jewel is found, the player must remove it (i.e., bring it outside the building using one of three possible exits) before searching for the next jewel. The participants were encouraged to collect as many jewels as possible, but they were never instructed at any time to recall the spatial layout of the building while playing the game.

In comparison, participants relegated to the second “directed navigator” group were explicitly taught the spatial layout of the entire building using AbES through a series of pre-determined paths and with the assistance of a sighted facilitator (Figure 2C). Training involved a complete step-by-step instruction of the building layout such that all the room locations, exits, and landmarks were encountered in a serial and repeated fashion along the interior perimeter (following a clockwise direction) similar to a “shoreline” exploration strategy. The paths followed were representative of a virtual recreation of an O&M lesson and the instructions given by a professional O&M instructor for the purposes of learning the spatial layout of the target building.

Finally, participants randomized to the third control group interacted with the AbES software under the same self-directed exploratory strategy as the gamer group. However, in contrast to the gamer group, the virtual environment explored did not correspond to the target physical building (Figure 2D). As with the gamer group, the control participants were never instructed to recall the spatial layout of the building while playing the game.

### Software

The AbES software was developed using C++ programming language with Visual Studio.NET and framework 2.0 on a PC computer (Windows XP/7 operating system). The software runs using a 10 Mb HD, 1 Gb RAM Pentium processor using a standard laptop computer and sound card. Based on the original architectural floor plan of an existing building (located at the Carroll Center for the Blind; Newton, MA, USA), the rendered indoor virtual environment includes 23 rooms, a series of connecting corridors, three separate entrances and two stairwells (see Figures 2B,C). This building was selected by design given that it is physically removed from the main campus facilities and was not normally accessible to the clients of the Center. The design specifics of this user-centered audio-based interface have been described in detail elsewhere (see Connors et al., 2013). Briefly, using simple keystrokes, the user-centered software allows an individual to explore the virtual environment (space bar for moving forward, “L” for right, “J” for left, and “K” to open doors) and survey the spatial layout of the building. The “F” key could also be used to identify the individual's location at any time. Scaling of the virtual environment is such that each virtual step approximates one step in the real physical building. While moving through the environment, contextual auditory and spatial information is acquired sequentially and is continuously updated, allowing the user to build a corresponding mental representation of the building's spatial layout. Spatial and situational information are based on iconic and spatialized sound cues provided after each step and updated to match the user's egocentric heading. For example, if a door is located on the user's right side, a door knocking sound is heard in the user's right ear. Conversely, if the user turns around 180° so that the same door is now located on their left side, the sound is heard in the left channel. Finally, if the user is facing the door, the same knocking sound is heard in both ears. Orientation is based on cardinal compass headings (e.g., “north” or “east”) and text to speech (TTS) is used to provide further information regarding a user's current location, orientation and heading (e.g., “you are in the corridor, on the first floor, facing west”) as well as the identity of objects and obstacles in their path (e.g., “this is a wall”). Distance cues are provided by modulating sound intensity (e.g., the sound of a nearby jewel increases as it is approached, pitch increases as the user walks up a flight of stairs). In this manner, the software plays an appropriate audio file as a function of the user's location and orientation and keeps track of their position as they move through the environment.

### Behavioral testing

All participants interacted with AbES for the same amount of time (total of 90 min spread over three training sessions) regardless of the group they were relegated to. Following game play/training, all participants underwent a series of three sequential behavioral task assessments. These tasks were designed to evaluate their ability to transfer the spatial information while navigating within the virtual representation and corresponding physical environment modeled in AbES. The target paths used in the navigation assessments were never explicitly taught to any of the groups during the training/game play period.

A series of stop rules were implemented using criteria determined from pilot testing and performance assessments carried out prior to commencing the study. First, subjects were not allowed more than 6 min to carry out any given route task. If the participant was unable to complete the route task in the allotted time, a score of zero was given and the full 6 min was scored as the time taken. This upper time limit was defined as twice the standard deviation (*SD*) collected from the mean navigation times observed during pilot testing (thus by definition, a time greater than 6 min would be interpreted as an outlier response). Second, subjects were required to complete at least three out of the first five tested paths (either successfully or unsuccessfully, but within the designated time limit) for a given series of navigation tasks in order to proceed with the entire behavioral evaluation. These stop rules served two purposes. First, setting an upper limit on exploration time helped ensure that performance would be comparable across runs, tasks, and individuals, thus allowing for a more direct comparison and statistical analysis of performance. Second, from an ethical standpoint, enforcing stop rules would ensure participants would not be required to continue if their initial performance on a given behavioral assessment was too poor. As these participants were viewed as psychologically at-risk, it was deemed crucial to maintain their overall well-being and remain vigilant to any situation that may be perceived as exacerbating their sense of failure or personal frustration. Apart from the individuals relegated to the control group (see results below), these criteria were met by the all the participants randomized to the gamer and directed navigator arms of the study.

#### Virtual navigation

In the virtual navigation task, participants were instructed to complete a series of 10 predetermined paths in the virtual environment modeled in AbES. The paths used were a series of start and stop locations (i.e., rooms) and were all of comparable length and complexity in terms of path length and number of turns. The start-stop location pairs were loaded into the AbES software and presented automatically following a randomized order for each subject. Task success and navigation time were automatically scored and data was output to a text file for further analysis. Primary outcome measures included whether the participant was able to successfully complete the 10 navigation routes (i.e., number of correct paths expressed as percent correct) and the time taken (seconds) to reach the target.

#### Physical navigation

Following the first task assessment, participants were then taken to the physical building modeled in the AbES software and navigation performance was again assessed using a series of 10 predetermined routes of comparable length and complexity. Similar to the previous task assessment, participants were instructed to navigate a set of 10 predetermined targets presented in random order. Navigation performance was recorded by an experienced investigator following behind the study participant. Using a stopwatch, timing commenced once the subject took their first step and stopped when the subject verbally reported that they were in front of the door of the target destination. Navigation success (number of correct paths expressed as percent correct) and time to target (seconds) were collected.

#### Drop off

Finally, for the drop off task, participants were placed at five predetermined locations and instructed to exit the building using the shortest path possible relative to their starting point. To successfully carry out this task, subjects had to mentally choose one out of three possible exits relative to their starting position and navigate the route leading to that exit. Paths were scored such that the shortest possible path was given maximum points (i.e., three for the shortest path, two for the second, one for the longest, and no points for not being able to complete the task in the time allotted). Navigation time (seconds) was also collected.

While there was no direct measure of chance performance for these navigation tasks, it is important to note that there were 23 possible destinations (target rooms) from any given starting point. Further, scoring was based on the participant's first verbal response (in the case of the physical navigation tasks) once they reported arriving to the intended target. This latter rule was to ensure that subjects could not change their response once it was given, or give multiple responses within the allotted time in the hopes of arriving to the correct destination. Feedback on participants' navigation performance was not provided during the assessments.

### Data analysis

All data were analyzed using SPSS statistical software package (IBM, version 20). Three-Way (2 × 2 × 2) ANOVAs including condition (game/navigators) × visual experience (early/late) × gender (male/female) were performed for each outcome measure. *Post-hoc* tests were performed between groups following tests for interaction. We report mean and *SD* values with a significance level set at *p* < 0.05. Measures of association between the primary outcome of interest (i.e., percentage correct on physical navigation task) and additional factors (i.e., age, and verbal memory ability) were calculated using the Pearson product-moment correlation coefficient.

## Results

Overall, all participants were able to successfully interact with the AbES software. Following the navigation assessments, subjects in both the gamer and directed navigator groups demonstrated performance consistent with positive near transfer of learning. Specifically, these subjects applied the spatial information they acquired to successfully carry out navigation tasks within the virtual as well as physical environment modeled in AbES.

In contrast, it is important to note that this transfer of learning was not evidenced in the participants (both early and late blind) relegated to the control group of the study. That is, following game play in a non-contextual environment, these two subjects were unable to complete any of the navigation tasks successfully. Specifically, all subjects failed to find any target destinations on the first five paths tested (and timed out on each run) on all three of the navigation task assessments (i.e., virtual, physical, and drop off). As these control participants were unable to carry out any of the navigation task assessments, we interpreted their performance following training in the control arm as being functionally zero.

### Comparing performance in gamers and directed navigators—arriving to target

Evidence of transfer of learning on all the navigation task assessments was observed in both the gamer and directed navigator groups as well as in both early and late blind participants.

As a first level of analysis, Three-Way ANOVAs (2 condition × 2 visual experience × 2 gender) were used to confirm the effectiveness of the randomization procedure across groups. There were no differences between groups in terms of age [condition: *F*_(1, 27)_ = 0.888, *p* = 0.357, η^2^_*p*_ = 0.041; visual experience: *F*_(1, 27)_ = 1.516, *p* = 0.232, η^2^_*p*_ = 0.067; gender: *F*_(1, 27)_ = 0.122, *p* = 0.730, η^2^_*p*_ = 0.006]. No significant differences in verbal memory ability (Wechsler score) were apparent between gamers and directed navigators [*F*_(1, 27)_ = 2.823, *p* = 0.108, η^2^_*p*_ = 0.119], as well as the interaction of condition × gender [*F*_(1, 27)_ = 3.4230, *p* = 0.087, η^2^_*p*_ = 0.133]. As expected, the early blind group had significantly more O&M experience than the late blind group [*F*_(1, 27)_ = 18.245, *p* < 0.001, η^2^_*p*_ = 0.465], but there was no statistical difference between gamers and directed navigators [*F*_(1, 29)_ = 0.549, *p* = 0.467, η^2^_*p*_ = 0.025].

**Figure 3:**
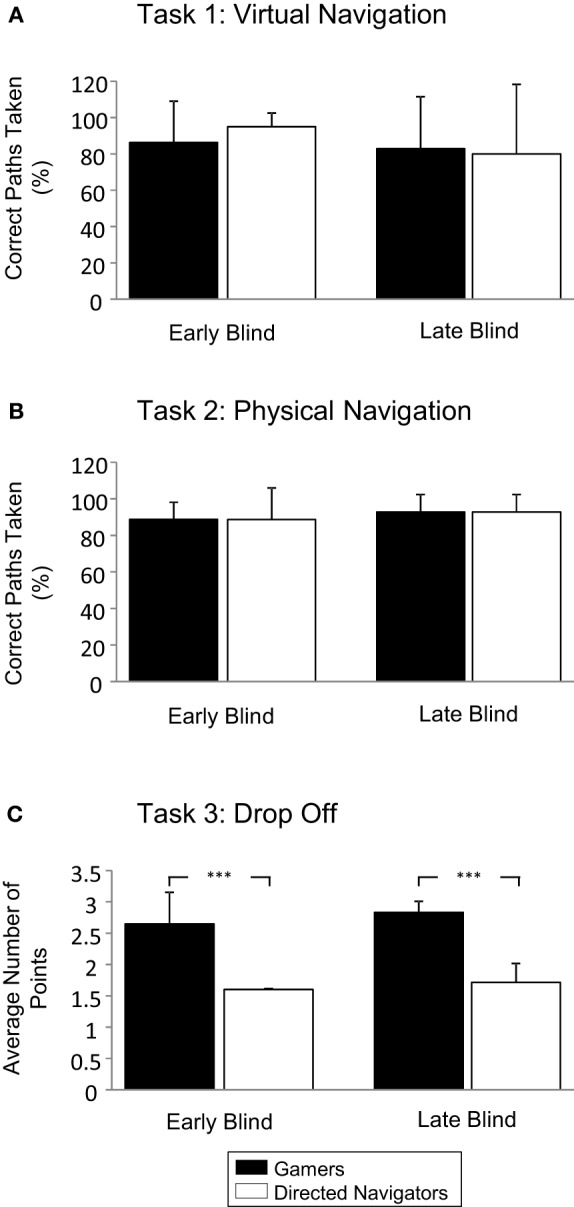
**Navigation performance—arriving to target**. Comparing performance on navigation tasks between gamers and directed navigator learning strategy in early and late blind participants. **(A)** High success on correct paths taken (%) for virtual room-to-room navigation was observed in both groups. **(B)** Similar high transfer success on correct paths taken (%) was observed for physical room-to-room navigation. **(C)** Results of the drop off task reveal an advantage for gamers. Paths chosen were scored such that the shortest route possible to exit the building from a given starting point received a maximum of 3 points, 2 for next closest exit, 1 for the longest, 0 for unsuccessful. Gamers showed an advantage over directed navigators in that they were more likely to choose the shortest path on the drop off task (indicated by higher average point score). The Three-Way ANOVA revealed a significant main effect for condition (see text). Error bars indicate SD, ^***^p < 0.001.

Comparing performance on virtual navigation task, a Three-Way ANOVA (2 condition × 2 visual experience × 2 gender) revealed no significant main effects for condition [*F*_(1, 27)_ = 0.231, *p* = 0.636, η^2^_*p*_ = 0.011], prior visual experience [*F*_(1, 27)_ = 1.283, *p* = 0.27, η^2^_*p*_ = 0.058], or gender [*F*_(1, 27)_ = 3.857, *p* = 0.063, η^2^_*p*_ = 0.155]. The interaction of condition and visual experience was not significant [*F*_(1, 25)_ = 0.064, *p* = 0.803, η^2^_*p*_ = 0.003], nor were any of the other interactions tested. Early blind gamers and directed navigators showed similar success on the navigation tasks following training with AbES (gamers: 85.00% ± 23.30 correct, directed navigators: 82.86% ± 9.51 correct; *p* = 0.942, n.s., Tukey test) (see Figure 3A). A similar profile of performance was observed in late blind gamers (82.86% ± 28.70) and directed navigators (80.00% ± 38.30) (*p* = 0.997, n.s., Tukey test). However, no significant difference was observed between gamers and directed navigators regardless of early and late blind status.

For the physical navigation task, a Three-Way ANOVA (2 condition × 2 visual experience × 2 gender) found no significant main effects for condition [*F*_(1, 27)_ = 0.070, *p* = 0.793, η^2^_*p*_ = 0.003], visual experience [*F*_(1, 27)_ = 1.761, *p* = 0.199, η^2^_*p*_ = 0.077], or gender [*F*_(1, 27)_ = 1.127, *p* = 0.301, η^2^_*p*_ = 0.051]. The interaction of condition and visual experience was not significant [*F*_(1, 25)_ = 0.070, *p* = 0.793, η^2^_*p*_ = 0.003], nor were any of the other interactions tested. Early blind gamers and directed navigators again showed comparable levels of performance in terms of their ability to transfer acquired spatial information to the real physical environment (gamers: 87.50% ± 10.35; directed navigators: 88.57% ± 18.64; *p* = 0.998, n.s., Tukey test). Similar performance levels were seen in late blind gamers and directed navigators as well (gamers: 92.86% ± 9.51; directed navigators: 92.86% ± 9.51; *p* = 1.00, n.s., Tukey test) (see Figure 3B). A repeated-measures ANOVA showed that the overall mean percentage correct performance on physical navigation (mean = 90.35% ± 12.10) was not statistically different from virtual navigation (mean=85.17% ± 25.86) [*F*_(1, 28)_ = 1.124, *p* = 0.298, η^2^_*p*_ = 0.039]. Thus, as with the virtual navigation task, no significant difference was observed between gamers and directed navigators regardless of early and late blind status.

Finally, assessing performance on the drop off task (i.e., exiting the building using the shortest path possible) did reveal a significant difference in performance between gamers and directed navigators. A Three-Way ANOVA (2 condition × 2 visual experience × 2 gender) found a significant main effect of condition [*F*_(1, 27)_ = 62.856, *p* < 0.001, η^2^_*p*_ = 0.750], but no main effect of visual experience [*F*_(1, 27)_ = 1.802, *p* = 0.194, η^2^_*p*_ = 0.079] or gender [*F*_(1, 27)_ = 0.003, *p* = 0.960, η^2^_*p*_ = 0.000]. The interaction of condition and visual experience was not significant [*F*_(1, 25)_ = 0.108, *p* = 0.745, η^2^_*p*_ = 0.005], nor were any of the other interactions tested. By group, the performance of the gamers (mean = 2.71 points ± 0.41 out of a maximum of 3) was significantly better than the directed navigators (mean = 1.66 points ± 0.21). Comparing performance between early blind gamers and directed navigators revealed effects similar to the overall group. On average, early blind gamers scored more points (2.60 points ± 0.52) than their directed navigator counterparts (1.60 points ± 0.00) (*p* < 0.001, Tukey test) suggesting that on average, gamers were more likely to select the closest exit and navigate using the shortest path regardless of their initial starting point. In contrast, directed navigators were more likely to use longer routes. A similar effect was seen in late blind gamers who scored on average 2.83 points ± 0.18 points, while directed navigators scored 1.71 points ± 0.30 (*p* < 0.001, Tukey test) (see Figure 3C).

### Comparing performance in gamers and directed navigators—time to target

**Figure 4:**
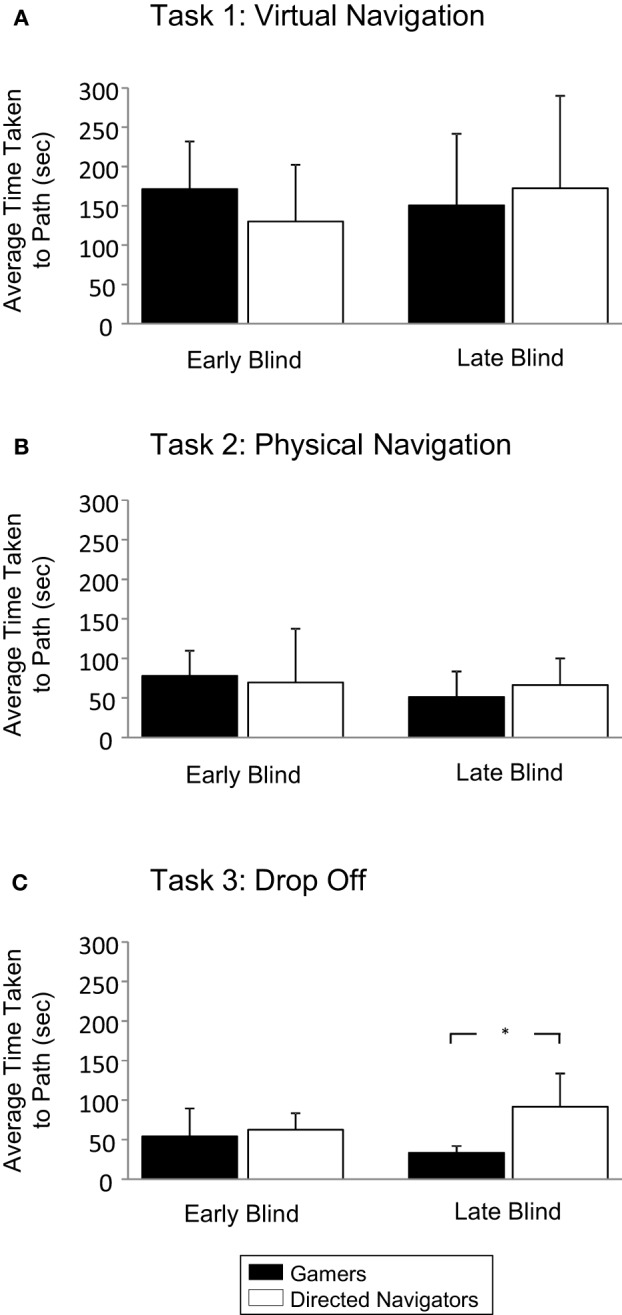
**Navigation performance—time to target**. Average time taken to navigate to target in the virtual navigation task **(A)** and the physical navigation task **(B)** was also similar across groups. **(C)** For the drop off task, gamers were generally faster than directed navigators to reach their target using an alternate route; however the difference was only significant in the late blind group. The Three-Way ANOVA revealed a significant main effect for condition (see text). Error bars indicate SD, ^*^p < 0.05.

Assessing time to target for the virtual navigation task was performed using a Three-Way ANOVA (2 condition × 2 visual experience × 2 gender). This analysis found no main effect of condition [*F*_(1, 27)_ = 0.164, *p* = 0.690, η^2^_*p*_ = 0.008], visual experience [*F*_(1, 27)_ = 0.251, *p* = 0.622, η^2^_*p*_ = 0.012], or gender [*F*_(1, 27)_ = 2.577, *p* = 0.125, η^2^_*p*_ = 0.109]. The interaction of condition and visual experience was not significant [*F*_(1, 25)_ = 0.206, *p* = 0.655, η^2^_*p*_ = 0.010], nor were any of the other interactions tested. Performance was again comparable in both early blind groups (gamers: 170.56 s ± 68.05; directed navigators: 136.00 s ± 75.47; *p* = 0.880, n.s., Tukey test). Similar performance in terms of time taken to target was found in late blind participants (gamers: 150.70 s ± 90.84; directed navigators: 172.34 s ± 117.44; *p* = 0.968, n.s., Tukey test) (see Figure 4A).

For time taken to target on the physical navigation task, a Three-Way ANOVA (2 condition × 2 visual experience × 2 gender) found no main effect of condition [*F*_(1, 27)_ = 0.377, *p* = 0.546, η^2^_*p*_ = 0.018], visual experience [*F*_(1, 27)_ = 1.112, *p* = 0.304, η^2^_*p*_ = 0.050], or gender [*F*_(1, 27)_ = 0.456, *p* = 0.507, η^2^_*p*_ = 0.021]. The interaction of condition and visual experience was not significant [*F*_(1, 25)_ = 0.420, *p* = 0.524, η^2^_*p*_ = 0.020], nor were any of the other interactions tested. Performance in early blind gamers (75.26 s ± 36.01) and directed navigators (71.34 s ± 73.39) was not significantly different between groups (*p* = 0.998, n.s., Tukey test). For late blind gamers (51.34 s ± 32.07) and directed navigators (66.47 s ± 33.50), no significant difference in mean time was found (*p* = 0.929, n.s., Tukey test). A repeated-measures ANOVA found that, the navigation times were markedly shorter for the physical navigation task (mean = 66.42 s ± 44.99) than for the virtual navigation task (mean = 157.94 s ± 85.61) [*F*_(1, 28)_ = 36.694, *p* < 0.001, η^2^_*p*_ = 0.567] (see Figure 4B).

Finally, comparing time to target on the drop off task, a Three-Way ANOVA (2 condition × 2 visual experience × 2 gender) found a significant main effect of condition [*F*_(1, 27)_ = 7.42, *p* = 0.013, η^2^_*p*_ = 0.261], but no main effect of visual experience [*F*_(1, 27)_ = 0.071, *p* = 0.792, η^2^_*p*_ = 0.003] or gender [*F*_(1, 27)_ = 0.000, *p* = 0.93, η^2^_*p*_ = 0.000]. The interaction between condition and visual experience was not statistically significant [*F*_(1, 25)_ = 3.429, *p* = 0.078, η^2^_*p*_ = 0.140]. None of the other interactions showed significant differences. Further analysis revealed that early blind gamers' mean time (51.23 s ± 42.36) compared to directed navigators (62.14 s ± 22.72) was not statistically significant (*p*= 0.916, n.s., Tukey test). However, the mean navigation time for late blind gamers (33.26 s ± 8.71) was significantly shorter than for directed navigators (91.66 s ± 41.79) (*p* = 0.013 Tukey test). A repeated-measures ANOVA found that the mean overall time (59.28 s ± 37.44) was not statistically different from the physical navigation task [*F*_(1, 28)_ = 0.635, *p* = 0.432, η^2^_*p*_ = 0.022] (see Figure 4C).

### Comparing performance with controls: arriving and time to target

All 7 of the control participants were unable to reach any of the target locations for the virtual, physical, and drop off tasks, yielding a mean percent correct path score of 0. Likewise, all participants reached the maximum time limit (360 s) for time to target. Because all participants performed at floor levels, there was no variance in the data, precluding the use of ANOVAs or *t-tests* to compare the experimental groups with the control group. Instead, we used the performance levels of the control group as a measure of chance, and compared the experimental groups against these values using 1-sample *t*-tests.

Examination of arrival at target yielded performance significantly greater than 0 in the gamer group for all three tasks [virtual: *t*_(14)_ = 13.006, *p* < 0.001; physical: *t*_(14)_ = 34.857, *p* < 0.001; drop-off: *t*_(14)_ = 25.811, *p* < 0.001; see Figure 3]. The directed navigator group also had performance significantly greater than 0 for the virtual [*t*_(13)_ = 11.706, *p* < 0.001], physical [*t*_(13)_ = 23.583, *p* < 0.001], and drop-off [*t*_(13)_ = 29.000, *p* < 0.001] navigation tasks (see Figure 3).

Time to target was compared against 360 s, the score for all participants in the control group. The gamer group was significantly faster than 360 s for the virtual [*t*_(14)_ = −9.971, *p* < 0.001], physical [*t*_(14)_ = −32.522, *p* < 0.001] and drop-off [*t*_(14)_ = −38.545, *p* < 0.001] tasks (see Figure 4). The directed navigator group was also significantly faster than 360 s for all three tasks [virtual: *t*_(13)_ = −7.961, *p* < 0.001; physical: *t*_(13)_ = −19.852, *p* < 0.001; drop-off: *t*_(13)_ = −29.620, *p* < 0.001; see Figure 4]. Thus, for both arrival and time to target, both the gamers and directed navigators were significantly above control performance for all three navigation tasks.

### Associations of interest

**Figure 5:**
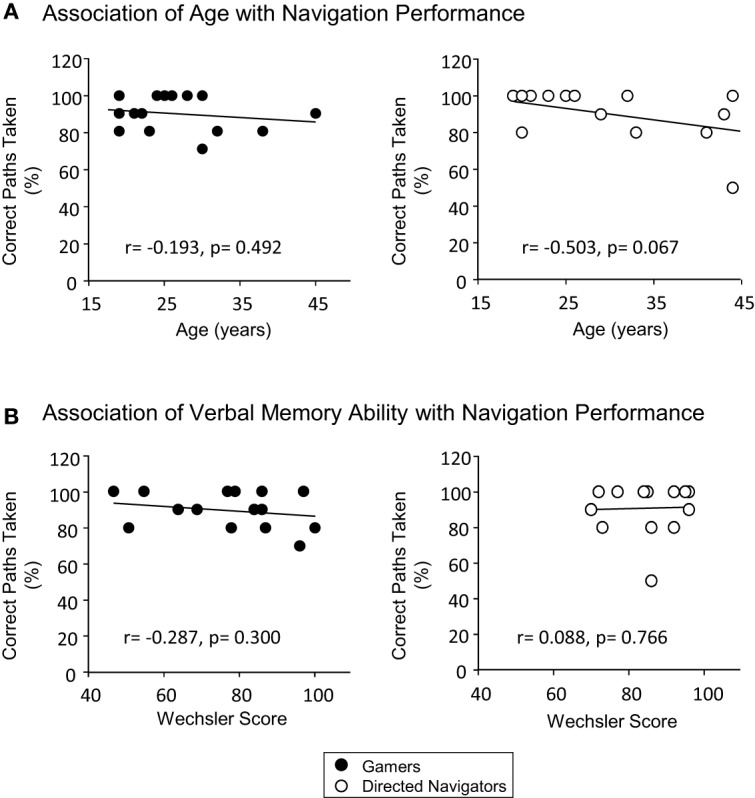
**Correlations between navigation task performance (% correct on physical navigation) and factors of interest (A) age and (B) verbal memory ability**. No significant associations were observed (data from gamers and directed navigators collapsed).

As a secondary analysis, we explored potential associations between navigation performance (assessed using percentage success on physical navigation as the primary outcome of interest) and the factors of age and verbal memory. Comparing individual navigation success with age in both conditions (collapsing early and late blind) revealed negative trends for both gamers [*r*_(13)_ = −0.193, *p* = 0.492] and directed navigators [*r*_(12)_ = −0.503, *p* = 0.067], although neither trend achieved statistical significance (Figure 5A). Similarly, no statistically significant association was evident comparing individual navigation performance with verbal memory recall (indexed by the Wechsler score) in either group [gamers: *r*_(13)_ = −0.287, *p* = 0.300; directed navigators: *r*_(12)_ = 0.088, *p* = 0.766] (Figure 5B). A second level analysis using a One-Way ANOVA with gender and group as factors confirmed a lack of association between navigation performance and gender for either the gamer [*F*_(1, 13)_ = 0.263, *p* = 0.617, η^2^_*p*_ = 0.020] or directed navigator group [*F*_(1, 12)_ = 1.751, *p* = 0.210, η^2^_*p*_ = 0.127]. Ancillary analyses exploring potential associations between the other assessments of navigation performance (i.e., virtual navigation and drop off task) with the factors of age and verbal memory revealed no further significant correlations.

## Discussion

In this study, we demonstrate that early and late blind individuals were able to effectively interact and explore an audio-based virtual environment for the purposes of acquiring relevant sensory information regarding a building's spatial layout. Furthermore, participants were able to generate a corresponding spatial cognitive map of the building they explored. The accuracy of this mental representation was confirmed by the fact that participants were able to successfully transfer acquired spatial information to a series of navigation tasks carried out in both the corresponding virtual environment explored and the physical building modeled in the AbES software. Furthermore, control subjects did not show any evidence of transfer. Specifically, the failure of the control subjects to carry out any of the navigation task assessments following game play suggests that a contextual overlap between the exploratory training and task environments is needed for the transfer of learning and further, game play alone cannot account for the behavioral performance observed.

In general, virtual and physical navigation performance was comparable whether participants learned the building's spatial layout implicitly through exploratory game play (gamers) or explicitly through structured and serial instructions (directed navigators). The similar performance between gamers and directed navigators suggests that both learning strategies (i.e., self-directed, implicit learning through gaming vs. guided instruction, explicit learning through directed navigation) allowed for the virtual exploration and subsequent generation of an accurate spatial representation that could be eventually transferred for the purposes of carrying out a large-scale and complex physical navigation task. As the target building contained two-stories, this also included successful navigation performance along the vertical dimension. This has been reported to be more difficult to achieve when acquiring information from virtual environments characterizing an indoor spatial layout (see Richardson et al., 1999, and later discussion). The most important difference between the two learning strategies compared was observed when the navigation task required the mental manipulation of this spatial information for determining an alternate route or short cut. Specifically, participants from the gamer group showed a behavioral advantage on the drop off task compared to the directed navigator group. Gamers were on average more likely to use the shortest route possible relative to their starting point compared to their directed navigator counterparts. These results suggest that training experience through gaming provided for a more flexible use of the spatial information characterizing the layout of the target building.

Navigation times were also comparable across groups for the virtual and physical navigation tasks. However, overall navigation time was shorter when these tasks were carried out in the physical compared to the virtual environment. This latter finding is consistent with anecdotal reports provided by a number of participants describing their impressions that the physical building was perceived to be smaller than what they had mentally imagined following the virtual exploration of the same environment. This anecdotal finding is somewhat puzzling given that the scale of the AbES environment was designed such that one step corresponded to one step in the physical building. The discrepancy may be related to inter-individual differences in how the resultant spatial cognitive map is generated or associated physical differences of the participants (such as individual stride length). Moreover, as virtual navigation always preceded physical navigation, there remains the possibility of a carryover effect arising from the sequential assessment of performance. Regarding the drop off task, navigation times for the gamers were faster than their directed navigator counterparts. This trend is consistent with the observation that participants on average chose the shortest possible route.

Finally, overall navigation performance did not appear to be strongly associated with other factors of interest including age, gender, and verbal memory skill.

The transfer of spatial knowledge from exploring virtual environments has been investigated previously under varied conditions in sighted subjects. In general, results from these studies have highlighted that immersion in a virtual environment can facilitate transfer of knowledge when the fidelity between the gaming environment and the real-world building is high (Waller et al., 1998; Farrell et al., 2003). The importance of contextual overlap between the learning and task settings is referred to as near transfer of learning (Cormier and Hagman, 1987). The results observed in this study can be viewed as consistent with this form of learning. This is also supported by the fact that the control group showed no evidence of transfer after game play in a virtual environment that did not correspond to the spatial layout of the target building. It is also worth highlighting that the gamer and directed navigator groups were never trained in the paths used in the behavioral assessments. This suggests that participants were not only able to acquire the spatial information needed to generate an accurate mental representation of the environment, but were also able to access and manipulate this information for the purposes of carrying out navigation tasks. It would be of considerable interest to investigate specifically the effect of prolonged training and game play (say, on the order of months) to see if enhanced performance (i.e., transfer of learning) would occur within other spatial related tasks or cognitive domains beyond the training context investigated here. In this direction, a specific longitudinal, long term, study incorporating a battery of pre and post-performance assessments would need to be carried out to answer this question conclusively.

In terms of rehabilitation and training for the blind, the use of virtual environments and game based learning strategies have been investigated in a number of studies (for applications including navigation and other forms of cognitive development such as short term memory and math skills; see Sánchez and Baloian, 2005; Sánchez and Maureira, 2007; Merabet and Sánchez, 2009; Afonso et al., 2010; Saenz and Sánchez, 2010; Lahav et al., 2011). Similar approaches have also been pursued for individuals with cognitive disabilities (Strickland, 1997; Salem et al., 2012) as well as for physical rehabilitation (Cho et al., 2012). Learning through a ludic-based approach could have specific benefits with regards to the transfer of spatial knowledge. Indeed, it has been proposed that game-based exploration of a virtual environment may have several potential advantages over those who are directed through a novel environment (Chrastil and Warren, 2012). First, they can make self-directed decisions about how and where they wish to navigate, which requires them to keep track of locations they have previously visited, and test theories about where they expect to be with every turn (Wilson et al., 1997; von Stulpnagel and Steffens, 2012). Second, exploring through game play may require additional mental manipulation of the spatial layout of the environment, which can be beneficial for learning (Bosco et al., 2004; Coluccia et al., 2007). For example, a gamer would need to understand that rooms that were on the right side of the hallway when traveling in one direction are now on the left side when traveling in the opposite direction. Third, improved attentional processing may allow gamers to attend and recall navigation-relevant features of the environment, even when not given explicit instructions to do so (Magliano et al., 1995; Taylor et al., 1999). Finally, the immersive nature of video game play offers gamers the possibility to experience travel in the environment from multiple perspectives and reference frames, which might prove useful when trying to determine how to reach a particular goal and manipulate information to find an alternate route.

In this study, participants in the directed navigator group served as a reference group to characterize performance associated with the transfer of information based on explicit training. It is of interest that participants in the gaming and directed navigator groups showed comparable levels of performance despite the fact that gamers were never explicitly told to retain any information regarding the spatial layout of the building, nor were they aware that the overall purpose of the study. Moreover, the gamer group showed an advantage over the directed navigation group in the drop-off task in that gamers were more likely to find shorter routes (likely as a result of being able to better mentally manipulate their resultant spatial map). As outlined earlier, it is possible that the highly engaging, interactive, and immersive nature of game play supported the development of more flexible and robust spatial cognitive constructs, particularly in situations where spatial information had to be manipulated for the purposes of determining optimal and alternative routes. We propose that the differences in learning strategy and subsequent effects on behavioral performance observed are likely related to the method through which spatial information is acquired, organized, and how the resultant spatial cognitive map is developed. The results of this study confirm and validate our initial proof-of-concept findings that learning through game play may provide for superior contextual learning and transfer of situational knowledge resulting from greater understanding of the spatial inter-relations within a building environment (Merabet et al., 2012). While individuals who learned the building environment implicitly through gaming exhibited a transfer of learning, those who learned the same environment in an explicitly guided manner appeared to fail in capturing more global contextual and situation-relevant information, such as where to exit the building. This somewhat constrained response profile exhibited by the latter group is consistent with what is typically seen following more rote type learning strategies (Shaffer et al., 2005).

The fact that participants showed high levels of success in transferring spatial information to real world navigation tasks suggests that the mental representations generated were isomorphic with the real world spatial layout of the target environment (Richardson et al., 1999; Waller et al., 2001). Given that visual cues play a crucial role in capturing and organizing spatial formation, it has been assumed that blind individuals (and in particular, those who are born with profound blindness) would be impaired in creating an accurate mental spatial representation of their surroundings (von Senden, 1960; Ashmead et al., 1989; Thinus-Blanc and Gaunet, 1997; see also Blasch et al., 1997 for further discussion). It is thus of interest that both early and late blind participants showed similar levels of performance in this study. Indeed, the effect of previous visual experience has long been an issue of debate not only in terms of teaching O&M skills, but also with regards to other spatial processing tasks (Cornoldi et al., 1991, 2009; Aleman et al., 2001; Vecchi et al., 2004; Tinti et al., 2006; Cattaneo et al., 2008). A review of this literature reveals contradictory results (particularly in relation to the role of prior visual experience), calling into question the validity of these earlier assumptions. In fact, some studies have reported that no differences exist in terms of how well blind individuals are able to mentally represent and interact with spatial environments (Landau et al., 1981; Passini and Proulx, 1988; Morrongiello et al., 1995; Noordzij et al., 2006) and in certain spatial navigation tasks, individuals with profound blindness have been shown to exhibit equal (Loomis et al., 2001) and in some cases, superior performance (Fortin et al., 2008) when compared to sighted control subjects.

In a recent and comprehensive review, Pasqualotto and Proulx (2012) discuss how visual development may be necessary for the normal capacities of spatial cognition. This argument is based on the ability of the visual modality to capture and convey parallel information, as well as to provide a basis for topographic representations necessary for high level (i.e., “allocentric”) spatial representations and multisensory integration (Pasqualotto and Proulx, 2012). In other words, spatial processing strategies and underlying representations may be different depending on the amount of prior visual experience available (see also Pasqualotto et al., 2013 for results demonstrating the preference of congenitally blind individuals in using egocentric based reference frames for the spatial representation of objects in space). In the present study, early and late blind participants exhibited comparable behavioral performance, suggesting that the immersive and exploratory nature of the virtual environment was sufficient to promote the accurate generation of a spatial cognitive map for the purposes of navigation. However, we are unable at this juncture to determine whether either group was preferentially employing strategies more aligned with egocentric or allocentric based representations. Furthermore, we acknowledge that while our early blind participants had documented evidence of profound blindness assessed prior to the age of three, disentangling the true effect of prior visual experience is less definitive based on the blindness history of the participants enrolled in this study. Finally, it is important to consider that overall behavioral performance may be related to not only the nature of the task demands, but also to the scale of the environment tested. At the same time, we recognize that early blind individuals typically will have spent more time learning compensatory strategies than late blind individuals, and thus the behavioral and associated neurophysiological adaptations at play may be very different between these two groups (Carroll, 1961). This remains a key question requiring careful study, as it has important implications not only in terms of understanding development and compensatory behavioral changes, but also for rehabilitation and educational strategies for the blind in general (Merabet and Pascual-Leone, 2010). A continuation of this study encompassing a larger scale and more complex mental map (e.g., incorporating indoor and outdoor environments) is needed to confirm whether there are indeed discrepancies related to visual experience and overall navigation performance.

Finally, no strong associations were apparent between navigation performance (as indexed by the percentage correct on the physical navigation task) and other factors of interest including age and verbal memory ability. While such an absence of statistical evidence certainly does not rule out the possibility of a true underlying association, it does suggest that such a simulation-game based learning strategy can be effective across a wide demographic profile. Furthermore, no apparent differences in performance were observed as a function of gender. Indeed, differences in navigation and other spatial cognitive skills in men and women have long been reported and debated (Maguire et al., 1999; Bosco et al., 2004; Coluccia and Louse, 2004; Wolbers and Hegarty, 2010). Again, it is possible that the navigation task undertaken here did not allow for differences to be revealed between male and female participants or, more plausibly, that the nature of the training and assessments were such that any inherent gender-related differences in the acquisition and overall transfer of spatial skills were not made apparent (see Feng et al., 2007 reporting the use of an action based video game to reduce gender related differences of performance in spatial cognition tasks).

In conclusion, the findings from this study demonstrate that the highly interactive and immersive nature of the AbES system is effective for the learning of novel environments in both early and late blind individuals through a mechanism akin to near transfer of skill learning. Furthermore, active game play provided a more flexible cognitive representation of the environment, allowing gamers to mentally manipulate spatial information for the purposes of finding alternate routes. This learning approach may serve as a useful tool to learn the spatial layout of large-scale, three-dimensional spaces, and to transfer that knowledge into real-world navigation tasks.

## Author contributions

Analyzed the data: Erin C. Connors, Elizabeth R. Chrastil, Lotfi B. Merabet. Designed the research: Lotfi B. Merabet. Collected data: Erin C. Connors, Lotfi B. Merabet. Contributed to writing the paper: Erin C. Connors, Elizabeth R. Chrastil, Jaime Sánchez, Lotfi B. Merabet.

### Conflict of interest statement

The authors declare that the research was conducted in the absence of any commercial or financial relationships that could be construed as a potential conflict of interest.

## References

[B1] Afonso A. Blum A. Katz B. F. Tarroux P. Borst G. Denis M. (2010). Structural properties of spatial representations in blind people: Scanning images constructed from haptic exploration or from locomotion in a 3-D audio virtual environment. Mem. Cogn. 38, 591–604 10.3758/MC.38.5.591 20551339

[B2] Aleman A. van Lee L. Mantione M. H. Verkoijen I. G. de Haan E. H. (2001). Visual imagery without visual experience: evidence from congenitally totally blind people. Neuroreport 12, 2601–2604 10.1097/00001756-200108080-00061 11496156

[B3] Ashmead D. H. Hill E. W. Talor C. R. (1989). Obstacle perception by congenitally blind children. Percept. Psychophys. 46, 425–433 10.3758/BF03210857 2813027

[B4] Bavelier D. Green C. S. Han D. H. Renshaw P. F. Merzenich M. M. Gentile D. A. (2011). Brains on video games. Nat. Rev. Neurosci. 12, 763–768 10.1038/nrn3135 22095065 PMC4633025

[B5] Bavelier D. Green C. S. Pouget A. Schrater P. (2012). Brain plasticity through the life span: learning to learn and action video games. Annu. Rev. Neurosci. 35, 391–416 10.1146/annurev-neuro-060909-152832 22715883

[B6] Blasch B. B. Wiener W. R. Welsh R. L. (eds.). (1997). Foundations of Orientation and Mobility, 2nd Edn. New York, NY: AFB Press

[B7] Bosco A. Longoni A. M. Vecchi T. (2004). Gender effects in spatial orientation: cognitive profiles and mental strategies. Appl. Cogn. Psychol. 18, 519–532 10.1002/acp.1000 20676381 PMC2909401

[B8] Carroll T. J. (1961). Blindness: What it is, What it Does, and How to Live with it, 1st Edn. Boston, MA: Little

[B9] Cattaneo Z. Vecchi T. Cornoldi C. Mammarella I. Bonino D. Ricciardi E. (2008). Imagery and spatial processes in blindness and visual impairment. Neurosci. Biobehav. Rev. 32, 1346–1360 10.1016/j.neubiorev.2008.05.002 18571726

[B10] Chebat D. R. Schneider F. C. Kupers R. Ptito M. (2011). Navigation with a sensory substitution device in congenitally blind individuals. Neuroreport 22, 342–347 10.1097/WNR.0b013e3283462def 21451425

[B11] Cho K. H. Lee K. J. Song C. H. (2012). Virtual-reality balance training with a video-game system improves dynamic balance in chronic stroke patients. Tohoku J. Exp. Med. 228, 69–74 10.1620/tjem.228.69 22976384

[B63] Chrastil E. R. Warren W. H. (2012). Active and passive contributions to spatial learning. Psychon. Bull. Rev. 19, 1–23 10.3758/s13423-011-0182-x 22083627

[B66] Coluccia E. Bosco A. Brandimonte M. A. (2007). The role of visuo-spatial working memory in map learning: new findings from a map drawing paradigm. Psychol. Res. 71, 359–372 10.1007/s00426-006-0090-2 16983581

[B12] Coluccia E. Louse G. (2004). Gender differences in spatial orientation: a review. J. Exp. Psychol. 24, 329–340 10.1016/j.jenvp.2004.08.006

[B13] Connors E. C. Yazzolino L. A. Sanchez J. Merabet L. B. (2013). Development of an audio-based virtual gaming environment to assist with navigation skills in the blind. J. Vis. Exp. 73 10.3791/50272 23568182 PMC3641639

[B14] Cormier S. M. Hagman J. D. (1987). Transfer of Learning: Contemporary Research Applications. San Diego, CA: Academic Press Inc.

[B15] Cornoldi C. Cortesi A. Preti D. (1991). Individual differences in the capacity limitations of visuospatial short-term memory: research on sighted and totally congenitally blind people. Mem. Cogn. 19, 459–468 10.3758/BF03199569 1956307

[B16] Cornoldi C. Tinti C. Mammarella I. C. Re A. M. Varotto D. (2009). Memory for an imagined pathway and strategy effects in sighted and in totally congenitally blind individuals. Acta Psychol. 130, 11–16 10.1016/j.actpsy.2008.09.012 19013547

[B17] Dede C. (2009). Immersive interfaces for engagement and learning. Science 323, 66–69 10.1126/science.1167311 19119219

[B61] Farrell M. J. Arnold P. Pettifer S. Adams J. Graham T. MacManamon M. (2003). Transfer of route learning from virtual to real environments. J. Exp. Psychol. Applied 9, 219–227 10.1037/1076-898X.9.4.219 14664673

[B18] Feng J. Spence I. Pratt J. (2007). Playing an action video game reduces gender differences in spatial cognition. Psychol. Sci. 18, 850–855 10.1111/j.1467-9280.2007.01990.x 17894600

[B19] Fortin M. Voss P. Lord C. Lassonde M. Pruessner J. Saint-Amour D. (2008). Wayfinding in the blind: larger hippocampal volume and supranormal spatial navigation. Brain 131(Pt 11), 2995–3005 10.1093/brain/awn250 18854327

[B20] Giudice N. A. Bakdash J. Z. Legge G. E. (2007). Wayfinding with words: spatial learning and navigation using dynamically updated verbal descriptions. Psychol. Res. 71, 347–358 10.1007/s00426-006-0089-8 16983582

[B21] Giudice N. A. Legge G. E. (2008). Blind navigation and the role of technology, in Engineering Handbook of Smart Technology for Aging, Disability, and Independence, eds Helal A. Mokhtari M. Abdulrazak B. (John Wiley and Sons), 479–500

[B22] Johnson L. A. Higgins C. M. (2006). A navigation aid for the blind using tactile-visual sensory substitution. Conf. Proc. IEEE Eng. Med. Biol. Soc. 1, 6289–6292 10.1109/IEMBS.2006.259473 17945950

[B23] Kalia A. A. Legge G. E. Roy R. Ogale A. (2010). Assessment of indoor route-finding technology for people with visual impairment. J. Vis. Impair. Blind. 104, 135–147 21869851 PMC3160142

[B24] Kuppersmith R. B. Johnston R. Jones S. B. Jenkins H. A. (1996). Virtual reality surgical simulation and otolaryngology. Arch. Otolaryngol. Head Neck Surg. 122, 1297–1298 10.1001/archotol.1996.01890240007002 8956738

[B25] Lahav O. Schloerb D. W. Srinivasan M. A. (2011). Newly blind persons using virtual environment system in a traditional orientation and mobility rehabilitation program: a case study. Disabil. Rehabil. Assist. Technol. 7, 420–435 10.3109/17483107.2011.635327 22112148

[B26] Landau B. Gleitman H. Spelke E. (1981). Spatial knowledge and geometric representation in a child blind from birth. Science 213, 1275–1278 10.1126/science.7268438 7268438

[B27] Lange B. Koenig S. Chang C. Y. McConnell E. Suma E. Bolas M. (2012). Designing informed game-based rehabilitation tasks leveraging advances in virtual reality. Disabil. Rehabil. 34, 1863–1870 10.3109/09638288.2012.670029 22494437

[B28] Long R. G. Giudice N. A. (2010). Establishing and maintaining orientation for orientation and mobility, in Foundations of Orientation and Mobility, 3rd ed. Vol. 1, *History and Theory*, eds Blasch B. B. Wiener W. R. Welch R. W. (New York, NY: American Foundation for the Blind), 45–62

[B29] Loomis J. M. Klatzky R. L. Golledge R. G. (2001). Navigating without vision: basic and applied research. Optom. Vis. Sci. 78, 282–289 10.1097/00006324-200105000-00011 11384005

[B30] Loomis J. M. Klatzky R. L. Golledge R. G. Cicinelli J. G. Pellegrino J. W. Fry P. A. (1993). Nonvisual navigation by blind and sighted: assessment of path integration ability. J. Exp. Psychol. Gen. 122, 73–91 10.1037/0096-3445.122.1.73 8440978

[B31] Loomis J. M. Marston J. R. Golledge R. G. Klatzky R. L. (2005). Personal guidance system for people with visual impairment: a comparison of spatial displays for route guidance. J. Vis. Impair. Blind. 99, 219–232 20054426 PMC2801896

[B67] Magliano J. P. Cohen R. Allen G. L. Rodrigue J. R. (1995). The impact of a wayfinder's goal on learning a new environment: Different types of spatial knowledge as goals. J. Environ. Psychol. 15, 65–75 10.1016/0272-4944(95)90015-2

[B32] Maguire E. A. Burgess N. O'Keefe J. (1999). Human spatial navigation: cognitive maps, sexual dimorphism, and neural substrates. Curr. Opin. Neurobiol. 9, 171–177 10.1016/S0959-4388(99)80023-3 10322179

[B33] Mayo M. J. (2009). Video games: a route to large-scale STEM education? Science 323, 79–82 10.1126/science.1166900 19119223

[B34] Merabet L. B. Connors E. C. Halko M. A. Sanchez J. (2012). Teaching the blind to find their way by playing video games. PLoS ONE 7:e44958 10.1371/journal.pone.0044958 23028703 PMC3446956

[B35] Merabet L. B. Pascual-Leone A. (2010). Neural reorganization following sensory loss: the opportunity of change. Nat. Rev. Neurosci. 11, 44–52 10.1038/nrn2758 19935836 PMC3898172

[B36] Merabet L. Sánchez J. (2009). Audio-based navigation using virtual environments: combining technology and neuroscience. AER J. 2, 128–137

[B37] Morrongiello B. A. Timney B. Humphrey G. K. Anderson S. Skory C. (1995). Spatial knowledge in blind and sighted children. J. Exp. Child Psychol. 59, 211–233 10.1006/jecp.1995.1010 7722435

[B38] Noordzij M. L. Zuidhoek S. Postma A. (2006). The influence of visual experience on the ability to form spatial mental models based on route and survey descriptions. Cognition 100, 321–342 10.1016/j.cognition.2005.05.006 16043169

[B39] Pasqualotto A. Proulx M. J. (2012). The role of visual experience for the neural basis of spatial cognition. Neurosci. Biobehav. Rev., 36, 1179–1187 10.1016/j.neubiorev.2012.01.008 22330729

[B40] Pasqualotto A. Spiller M. J. Jansari A. S. Proulx M. J. (2013). Visual experience facilitates allocentric spatial representation. Behav. Brain Res. 236, 175–179 10.1016/j.bbr.2012.08.042 22960256

[B41] Passini R. Proulx G. (1988). Wayfinding without vision. Environ. Behav. 20, 227–252 10.1177/0013916588202006

[B42] Pataki C. Pato M. T. Sugar J. Rizzo A. S. Parsons T. D. St George C. (2012). Virtual patients as novel teaching tools in psychiatry. Acad. Psychiatry 36, 398–400 10.1176/appi.ap.10080118 22983473

[B43] Petrie H. Johnson V. Strothotte T. Raab A. Fritz S. Michel R. (1996). Mobic: designing a travel aid for blind and elderly people. J. Navigation 49, 45–52 10.1017/S0373463300013084

[B44] Richardson A. E. Montello D. R. Hegarty M. (1999). Spatial knowledge acquisition from maps and from navigation in real and virtual environments. Mem. Cogn. 27, 741–750 10.3758/BF03211566 10479831

[B45] Rizzo A. Buckwalter J. G. John B. Newman B. Parsons T. Kenny P. (2012). STRIVE: stress resilience in virtual environments: a pre-deployment VR system for training emotional coping skills and assessing chronic and acute stress responses. Stud. Health Technol. Inform. 173, 379–385 22357022

[B46] Saenz M. Sánchez J. (2010). Indoor orientation and mobility for learners who are blind. Stud. Health Technol. Inform. 154, 165–170 20543291

[B47] Salem Y. Gropack S. J. Coffin D. Godwin E. M. (2012). Effectiveness of a low-cost virtual reality system for children with developmental delay: a preliminary randomised single-blind controlled trial. Physiotherapy 98, 189–195 10.1016/j.physio.2012.06.003 22898574

[B48] Sánchez J. Baloian N. (2005, June 27–July 2). Modeling audio-based virtual environments for children with visual disabilities, in Paper Presented at the Proceedings of the World Conference on Educational Multimedia, Hypermedia and Telecommunications ED-MEDIA, (Montreal, QC).

[B49] Sánchez J. Maureira E. (2007). Subway mobility assistance tools for blind users, in Lecture Notes in Computer Science, LNCS, 4397, eds Stephanidis C. Pieper M. (New York, NY: Springer), 386–404

[B50] Shaffer D. W. Squire K. R. Halverson R. Gee J. P. (2005). Video games and the future of learning. Phi Delta Kappan 87, 104–111

[B51] Siegel A. W. White S. H. (1975). The development of spatial representations of large-scale environments. Adv. Child Dev. Behav. 10, 9–55 10.1016/S0065-2407(08)60007-5 1101663

[B52] Strelow E. R. (1985). What is needed for a theory of mobility: direct perception and cognitive maps–lessons from the blind. Psychol. Rev. 92, 226–248 10.1037/0033-295X.92.2.226 3887451

[B53] Strickland D. (1997). Virtual reality for the treatment of autism. Stud. Health Technol. Inform. 44, 81–86 10184809

[B68] Taylor H. A. Naylor S. J. Chechile N. A. (1999). Goal-specific influences on the representation of spatial perspective. Mem. Cognit. 27, 309–319 10.3758/BF03211414 10226440

[B54] Thinus-Blanc C. Gaunet F. (1997). Representation of space in blind persons: vision as a spatial sense? Psychol. Bull. 121, 20–42 10.1037/0033-2909.121.1.20 9064698

[B55] Tinti C. Adenzato M. Tamietto M. Cornoldi C. (2006). Visual experience is not necessary for efficient survey spatial cognition: evidence from blindness. Q. J. Exp. Psychol. 59, 1306–1328 10.1080/17470210500214275 16769626

[B56] Vecchi T. Tinti C. Cornoldi C. (2004). Spatial memory and integration processes in congenital blindness. Neuroreport 15, 2787–2790 15597055

[B57] von Senden M. (1960). Space and sight: the Perception of Space and Shape in the Congenitally Blind Before and After Operation. Free Press

[B64] von Stulpnagel R. Steffens M. C. (2012). Can active navigation be as good as driving? A comparison of spatial memory in drivers and backseat drivers. J. Exp. Psychol. Appl. 18, 162–177 10.1037/a0027133 22329733

[B58] WAIS-III W. M. S. (1997). Wechsler Memory Scale, Third Edition (WAIS-III) World List Test. Cleveland, OH: The Psychological Corporation

[B62] Waller D. Hunt E. Knapp D. (1998). The transfer of spatial knowledge in virtual environment training. Presence 7, 129–143

[B59] Waller D. Knapp D. Hunt E. (2001). Spatial representations of virtual mazes: the role of visual fidelity and individual differences. Hum. Factors 43, 147–158 10.1518/001872001775992561 11474760

[B65] Wilson P. N. Foreman N. Stanton D. (1997). Virtual reality, disability and rehabilitation. Disabil. Rehabil. 19, 213–220 10.3109/09638289709166530 9195138

[B60] Wolbers T. Hegarty M. (2010). What determines our navigational abilities? Trends Cogn. Sci. 14, 138–146 10.1016/j.tics.2010.01.001 20138795

